# Health Care Resource Use and Costs After Hospitalization With Multiple Organ Dysfunction in Children

**DOI:** 10.1001/jamanetworkopen.2024.56246

**Published:** 2025-01-29

**Authors:** Folafoluwa O. Odetola, Paul Lin, Wen Ye, Kevin J. Dombkowski, Ariel Linden

**Affiliations:** 1 Division of Pediatric Critical Care Medicine, Department of Pediatrics, University of Michigan, Ann Arbor; 2 Child Health Evaluation and Research Center, University of Michigan, Ann Arbor; 3 Institute for Health Policy and Innovation, University of Michigan, Ann Arbor; 4 Department of Biostatistics, School of Public Health, University of Michigan, Ann Arbor; 5 Department of Medicine, University of California, San Francisco

## Abstract

**Importance:**

Multiple organ dysfunction (MOD) is a leading cause of in-hospital child mortality. For survivors, posthospitalization health care resource use and costs are unknown.

**Objective:**

To evaluate longitudinal health care resource use and costs after hospitalization with MOD in infants (aged <1 year) and children (aged 1-18 years).

**Design, Setting, and Participants:**

This retrospective cohort study used nationwide data from 2004 to 2019 from Optum’s deidentified Clinformatics Data Mart Database, an insurance claims database. Infants and children from birth to age 18 years with an index hospitalization between January 1, 2005, and December 31, 2018, were included. Infants (age <1 year) and children (age 1-18 years) with MOD (MOD cohort) or without MOD (non-MOD cohort) were separately identified, and cohorts were propensity score weighted to balance demographics and pre–index hospitalization characteristics, including health care use and comorbidities. The data were analyzed between January 7, 2022, and September 8, 2023.

**Main Outcomes and Measures:**

Weighted generalized estimating equations were used to evaluate differences between cohorts in rehospitalizations, emergency department visits, and health care costs up to 5 years after the index hospitalization.

**Results:**

During the study period, 9671 children in the MOD cohort were compared with 1 691 793 children in the non-MOD cohort in the weighted sample. Infants comprised 67.4% of the MOD cohort (mean [SD] age at index hospitalization, 0.1 [0.8] years; 51.2% male) and 87% of the non-MOD cohort (mean [SD] age at index hospitalization, 0.1 [0.8] years; 50.8% male), and children comprised 32.5% of the MOD cohort (mean [SD] age at index hospitalization, 11.6 [5.7] years; 50.7% female) and 13.0% of the non-MOD cohort (mean [SD] age at index hospitalization, 11.5 [5.5] years; 51.3% female). The infant MOD cohort had more emergency department visits, with an adjusted incidence rate ratio of 1.76 (95% CI, 1.56-1.97) at 30 days; this difference persisted for years 1 through 5. Children had a similar pattern except at 30 days among those who acquired new organ-supportive technology during the index hospitalization. Among infants, the MOD cohort had more rehospitalizations, with an adjusted incidence rate ratio of 12.45 (95% CI, 11.40-13.59) at 30 days; this difference persisted for years 1 through 5. A similar pattern was observed among children. Annual health care costs were higher for the MOD cohort in year 1 (infants: mean [SD], $80 133 [$6543] vs $5183 [$19] [*P* < .001]; children: mean [SD], $54 113 [$17 544] vs $10 935 [$95] [*P* < .001]) and in all years through year 5.

**Conclusions and Relevance:**

In this cohort study of nearly 1.7 million children, survivors of MOD accrued substantial ongoing health care resource use and cost burden after the index hospitalization. These findings suggest that follow-up care of survivors of MOD should include economic well-being alongside measures of clinical health.

## Key Points

**Question:**

What are the health resource use and costs after an index hospitalization with multiple organ dysfunction (MOD) in infants (aged <1 year) and children (aged 1-18 years) compared with those without MOD?

**Findings:**

In this cohort study of 1 701 521 infants and children, those with prior hospitalization with MOD had significantly more rehospitalizations and higher health care costs over time than those without MOD. There were more emergency department visits overall among the MOD cohort except among older children at 30 days after the index hospitalization.

**Meaning:**

These findings suggest that follow-up care of survivors of MOD should include economic well-being alongside measures of clinical health.

## Teaser

This cohort study evaluates the longitudinal health care resource use and costs after an index hospitalization for multiple organ dysfunction in infants and children.

## Introduction

Multiple organ dysfunction (MOD) syndrome, the concurrent dysfunction of 2 or more organ systems,^1,2^ is a major cause of in-hospital child death and a harbinger of morbidity among children who survive critical illness and injury.^2,3,4,5,6,7,8,9,10^ The syndrome can result from severe critical illness, such as sepsis^3,4,8,11,12,13^; trauma^14,15,16,17^; surgery^18^; and from therapies, including solid organ transplant^19^ and hematopoietic stem cell transplant.^20,21^

Although medical advances have contributed to a substantial decline in child mortality from critical illness and injury from 20% in 1982^22^ to 1% to 5% in 2012,^23^ several studies have reported very low mortality in children without MOD,^2,24,25^ emphasizing the impact of MOD on child health outcomes. While prior reports have documented substantial in-hospital child mortality attributable to MOD,^2,10,22,24,25^ there is a paucity of information regarding the long-term health care resource use and costs among survivors of MOD. This area of inquiry is important because the life span and quality of life of MOD survivors could be severely compromised by premature mortality or persistent disease burden and morbidity, respectively, long after the initial hospitalization for critical illness.^26^ An enhanced understanding of this persistent burden, hitherto understudied, may provide valuable insights to clinicians and raise awareness of the need to not only prevent mortality but also mitigate long-term morbidity and economic burden. To test the hypothesis that children with MOD during an index hospitalization may have greater health care resource use than children without MOD, we evaluated the longitudinal health care resource use and economic burden between a cohort of children with MOD and a comparable cohort without MOD.

## Methods

### Data Source

For this retrospective, longitudinal cohort study, data were obtained from Optum’s deidentified Clinformatics Data Mart Database (CDM) (2001-2021), a large, integrated, geographically diverse US health insurance claims database covering 15 million lives in all states annually. The database has been previously used for other longitudinal studies.^27,28^ As data are deidentified, the University of Michigan Institutional Review Board deemed the study exempt from review and the need for informed consent. This report followed the Strengthening the Reporting of Observational Studies in Epidemiology (STROBE) reporting guideline.

The CDM was queried to obtain relevant inpatient and outpatient information, including demographic, diagnostic, therapeutic, pharmacy, cost, and outcomes data, using appropriate codes. All claims were linked using unique member identifiers and arrayed in chronologic order to provide a detailed longitudinal profile of all medical and pharmacy services used by each patient.

### Study Cohorts

Patients younger than 18 years who had an index hospitalization between January 1, 2005, and December 31, 2018, were included. Given reported differences in predisposition to MOD and overall subsequent survival by age,^7,29^ all analyses were conducted separately for infants (aged <1 year) and children (aged 1-18 years). The study period was chosen to provide 1 year of baseline data before the index hospitalization (lookback period) and at least 1 year of follow-up data for all patients. Infants, being younger than 1 year, could not have a full year of baseline data; however, infants in both cohorts had similar lookback periods. A cohort of patients with MOD (dysfunction of ≥2 organ systems) during the index hospitalization (MOD cohort) was identified using *International Classification of Diseases, Ninth Revision, Clinical Modification* (*ICD-9-CM*) and *International Statistical Classification of Diseases, Tenth Revision, Clinical Modification* (*ICD-10-CM*) diagnosis and procedure codes, as previously described^6,30,31^ (eAppendix 1 in Supplement 1). Patients hospitalized during the same study period who did not acquire MOD (no organ system dysfunction) during the index hospitalization and with an appropriate lookback period were selected as a comparison cohort (non-MOD cohort). Patients with 1 dysfunctional organ system were excluded from the analysis due to an inability to determine resolution or progression, which could bias the findings. Comorbidities were identified in the year prior to the index hospitalization using *ICD-9-CM* or *ICD-10-CM* codes via previously validated algorithms^32,33^ (eAppendix 2 in Supplement 1). Strict criteria were applied to ensure that there was no overlap between codes used to identify organ dysfunction and comorbidities in the year preceding the index hospitalization. The data source had no specific location code for intensive care unit (ICU) settings.

### Study Variables

Data were collected and analyzed relevant to specific time points. For the prehospitalization time point, patient characteristics and health care resource use captured within 1 year of the index hospitalization were used for balancing the cohorts on patient comorbidities^32,33,34,35^ and measures of prior inpatient and outpatient health care use and costs, which included standardized prior health care costs, number of office visits, number of laboratory tests ordered and number and mean duration of prior hospitalizations. During the index hospitalization, patient characteristics were captured for balancing of the cohorts on patient demographics, including age, race and ethnicity, sex, and state of residence.

For the posthospitalization time point, primary and secondary study outcomes were measured longitudinally after the index hospitalization. Primary outcomes included the number of emergency department (ED) visits at 30 days, 1 to 12 months, and 1 to 5 years; number of all-cause rehospitalizations at 30 days, 1 to 12 months, and 1 to 5 years; and total standardized annual costs of care at 1 to 5 years. Two secondary outcomes were also examined. The first was ongoing use of organ-supportive medical technology acquired during the index hospitalization, determined at 30 days, 1 to 12 months, and 1 to 5 years after discharge from the index hospitalization. The technology included dialysis, gastrostomy, nasal or oral enteral feeding tubes, tracheostomy, cerebral ventricular shunt devices, and central venous access devices. Dependence on technology beyond the index hospitalization served as a proxy for ongoing organ dysfunction. The other secondary outcome was use of health care services at 30 days, 1 to 12 months, and 1 to 5 years, including rehabilitative (physical, occupational, and speech) therapy services and home health care services.

### Propensity Score Weighting of Cohorts

To ensure comparability between the MOD and non-MOD cohorts and permit unbiased assessment of the study outcomes, inverse probability of treatment (IPT) weighting, a propensity score method,^36,37^ was used to adjust for systematic differences in baseline demographics and prehospitalization characteristics for up to 365 days prior to the index hospitalization between the cohorts. The propensity score was estimated as the probability of being assigned to the MOD cohort, conditional on baseline demographic characteristics and resource use in the year prior to the index hospitalization.^38,39^ For each child, the cohort assignment (MOD or non-MOD) was regressed on observed covariates in a logistic regression model.

The covariates incorporated into the model included age at the index hospitalization, categories of race and ethnicity (Asian, Black, Hispanic, White, and unknown) as reported in the CDM, categories of sex (female, male), categories of census division (East North Central, East South Central, Middle Atlantic, Mountain, New England, Pacific, South Atlantic, West North Central, West South Central, and unknown), measures of prior inpatient and outpatient health care use and costs, and comorbidities. Importantly, the length of the lookback period (in days) was included in the propensity score weighting for infants but not for children, as children had to have 365 days of lookback to be included in the cohort.

All participants were continuously enrolled in their insurance plan throughout each year of the study period to permit longitudinal assessment of health care resource use and were followed up for up to 5 years from the date of discharge from the index hospitalization until December 31, 2019, or until disenrollment from the health plan, whichever occurred first. Longitudinal data were evaluated with repeated measurement of the number of rehospitalizations, the number of ED visits, and costs.

### Statistical Analysis

The data analysis was performed between January 7, 2022, and September 8, 2023. Data were summarized by reporting mean (SD) or median (IQR) values for continuous variables and frequencies and percentages for categorical variables. Standardized mean differences were used to evaluate differences in baseline characteristics between the cohorts (both unweighted and IPT weighted), with a value less than 0.1 considered a small difference between cohorts.^40,41^ Bivariate comparisons of outcomes between cohorts were performed using weighted *t* test and Kruskal-Wallis test for continuous data with normal and nonparametric distribution, respectively. Comparison of categorical data was performed with weighted χ^2^ test with Rao-Scott second-order correction or weighted Pearson test, as appropriate. For ED visits and rehospitalizations, IPT-weighted multivariable regression models were estimated to adjust for newly acquired organ-supportive technology that remained after discharge from the index hospitalization and the use of rehabilitative therapy services after the index hospitalization, both factors that could trigger ED visits and rehospitalizations. Regression models also included an interaction term to assess whether ED visits and rehospitalizations differed by ongoing use of indwelling organ-supportive technology beyond the index hospitalization. For health care costs, multivariable regression models were estimated to control for acquired organ-supportive technology during the index hospitalization.

#### ED Visits and Rehospitalizations

Weighted generalized linear models, either zero-inflated Poisson or negative binomial regression models, were estimated based on dispersion of the outcome to compare the number of ED visits and rehospitalizations per patient between the 2 study cohorts at 30 days and 1 to 12 months. Weighted generalized estimating equations were estimated to compare the number of ED visits and rehospitalizations annually for up to 5 years after the index hospitalization. Between-cohort differences are reported as incidence rate ratios (IRRs) with their 95% CIs.

#### Costs

Standardized mean costs were adjusted to 2019 dollars to ensure comparability across study years. Weighted generalized estimating equations with γ-distribution were used to account for time trends in total costs between the 2 study cohorts and for clustering at the patient level.^42^ Using similar modeling approaches, the study cohorts were compared on their total standardized annual costs monthly for the first year and then annually for up to 5 years.

All data analyses were conducted using SAS, version 9 (SAS Institute Inc). There was no statistical adjustment for multiple comparisons given the exploratory nature of the study.^43,44^ Two-sided hypothesis testing was performed, with the threshold for statistical significance set at *P* < .05.

## Results

### Baseline Characteristics

**Figure 1.**
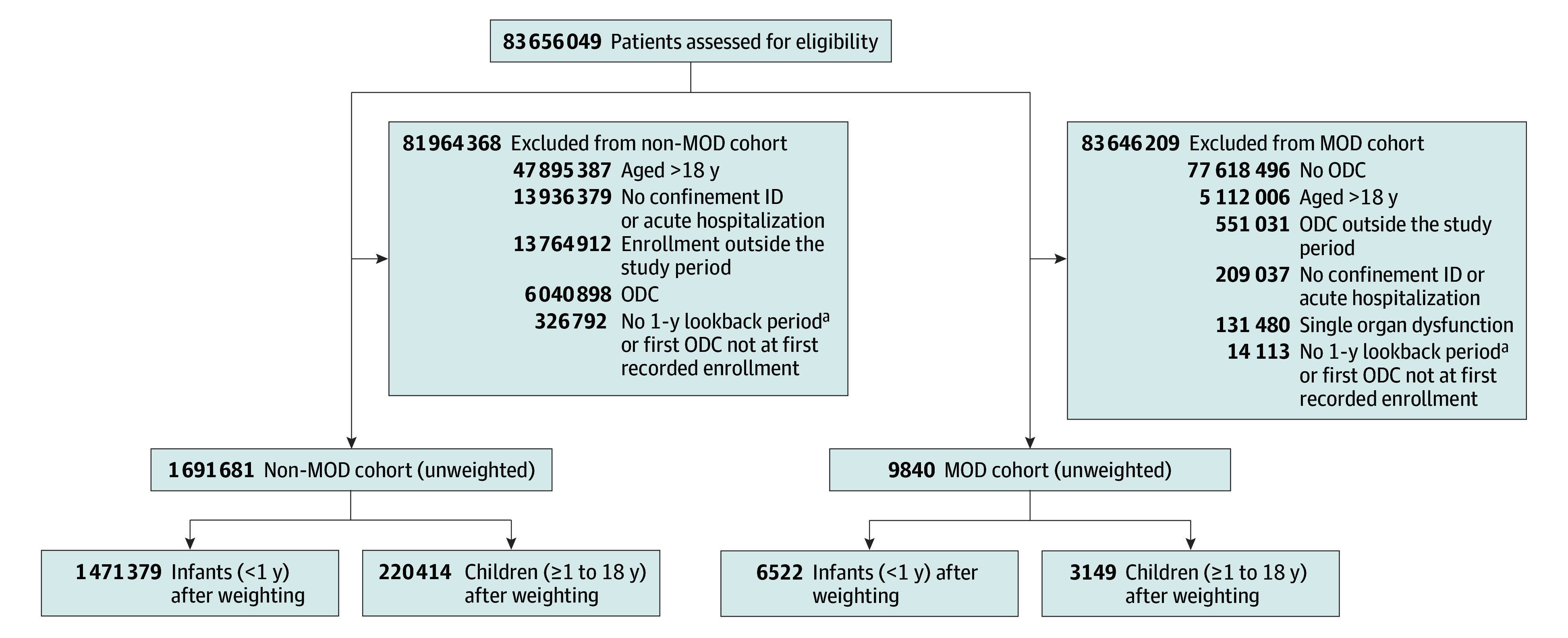
Flow Diagram of Cohort Selection ID indicates identifier; MOD, multiple organ dysfunction; ODC, organ dysfunction code. ^a^Except if aged 1 year or younger at index hospitalization.

During the study period, 9671 infants and children in the MOD cohort were compared with 1 691 793 infants and children in the non-MOD cohort in the weighted sample (Figure 1). Infants younger than 1 year accounted for 67.4% of the MOD cohort (6522 individuals; mean [SD] age at index hospitalization, 0.1 [0.8] years; 48.8% female and 51.2% male; 4.5% Asian, 4.7% Black, 6.9% Hispanic, 42.2% White, and 41.7% unknown race and ethnicity) and 87.0% of the non-MOD cohort (1 471 379 individuals; mean [SD] age at index hospitalization, 0.1 [0.8] years; 49.2% female and 50.8% male; 4.8% Asian, 4.5% Black, 6.9% Hispanic, 42.5% White, and 41.4% unknown race and ethnicity). Children aged 1 to 18 years accounted for 32.5% of the MOD cohort (3149 individuals; mean [SD] age at index hospitalization, 11.6 [5.7] years; 50.7% female and 49.3% male; 3.1% Asian, 8.5% Black, 10.5% Hispanic, 62.0% White, and 15.9% unknown race and ethnicity) and 13.0% of the non-MOD cohort (220 414 individuals; mean [SD] age at index hospitalization, 11.5 [5.5] years; 51.3% female and 48.7% male; 3.2% Asian, 8.6% Black, 10.9% Hispanic, 61.3% White, and 16.0% unknown race and ethnicity). Good balance was achieved between cohorts on all baseline patient demographics, comorbidities, and health care use in the year preceding the index hospitalization (Table 1).

**Table 1.** Baseline Characteristics of the Study Cohorts

Variable	Infants (aged <1 y), weighted No. (%)		SMD	Children (aged 1-18 y), weighted No. (%)		SMD
	MOD cohort (n = 6522)	Non-MOD cohort (n = 1 471 379)		MOD cohort (n = 3149)	Non-MOD cohort (n = 220 414)	
Sex						
Female	3185 (48.8)	723 856 (49.2)	0.01	1598 (50.7)	113 026 (51.3)	0.01
Male	3337 (51.2)	747 524 (50.8)		1551 (49.3)	107 388 (48.7)	
Race and ethnicity						
Asian	295 (4.5)	70 061 (4.8)	0.06	99 (3.1)	7081 (3.2)	0.04
Black	304 (4.7)	65 746 (4.5)		267 (8.5)	18 947 (8.6)	
Hispanic	450 (6.9)	100 973 (6.9)		331 (10.5)	24 052 (10.9)	
White	2750 (42.2)	625 107 (42.5)		1951 (62.0)	135 162 (61.3)	
Unknown	2721 (41.7)	609 492 (41.4)		501 (15.9)	35 172 (16.0)	
Census division						
East North Central	956 (14.7)	223 099 (15.2)	0.03	478 (15.2)	33 623 (15.3)	0.04
East South Central	218 (3.3)	51 149 (3.5)		121 (3.8)	8503 (3.9)	
Middle Atlantic	516 (7.9)	117 173 (8.0)		233 (7.4)	15 304 (6.9)	
Mountain	693 (10.6)	154 781 (10.5)		303 (9.6)	20 400 (9.3)	
New England	178 (2.7)	39 517 (2.7)		105 (3.3)	6946 (3.2)	
Pacific	568 (8.7)	129 826 (8.8)		282 (9.0)	19 759 (9.0)	
South Atlantic	1570 (24.1)	342 248 (23.3)		719 (22.8)	52 691 (23.9)	
West North Central	737 (11.3)	167 496 (11.4)		375 (11.9)	26 259 (11.9)	
West South Central	1070 (16.4)	243 257 (16.5)		527 (16.7)	36 506 (16.6)	
Unknown	14 (0.2)	2833 (0.2)		6 (0.2)	423 (0.2)	
Age at index hospitalization, mean (SD), y	0.1 (0.8)	0.1 (0.8)	0.04	11.6 (5.7)	11.5 (5.5)	0.02
Lookback, mean (SD), d^a^	4.16 (26.80)	3.14 (24.88)	0.04	NA	NA	NA
Standard cost prior to index hospitalization, mean (SD), $	495.08 (6143.82)	284.54 (5909.33)	0.04	9041.73 (28 306.69)	7688.28 (27 350.66)	0.05
No. of prior hospitalizations, mean (SD)	0.03 (0.19)	0.02 (0.17)	0.06	0.16 (0.56)	0.13 (0.55)	0.04
Duration of prior hospitalization, mean (SD), d	0.12 (0.96)	0.09 (0.95)	0.03	0.66 (3.22)	0.60 (4.19)	0.02
No. of office visits, mean (SD)	0.11 (0.96)	0.08 (0.82)	0.03	4.68 (5.45)	4.75 (4.73)	0.02
No. of ordered laboratory tests, mean (SD)	0.12 (1.58)	0.07(1.32)	0.04	7.14 (17.96)	7.50 (13.71)	0.02
Comorbidities in the year prior to the index hospitalization						
Neurologic or neuromuscular	7 (0.1)	868 (0.1)	0.02	97 (3.1)	6572 (3.0)	0.01
Cardiovascular	12 (0.2)	1830 (0.1)	0.02	118 (3.7)	7504 (3.4)	0.02
Respiratory	7 (0.1)	693 (0.0)	0.02	17 (0.5)	1264 (0.6)	0.00
Kidney or urologic	8 (0.1)	1027 (0.1)	0.02	46 (1.5)	2798 (1.3)	0.02
Gastrointestinal	8 (0.1)	713 (0.0)	0.02	48 (1.5)	3685 (1.7)	0.01
Hematologic or immunologic	3 (0.0)	526 (0.0)	0.01	47 (1.5)	3135 (1.4)	0.01
Metabolic	7 (0.1)	1181 (0.1)	0.01	64 (2.0)	4584 (2.1)	0.00
Other congenital or genetic anomaly	9 (0.1)	1822 (0.1)	0.01	142 (4.5)	10006 (4.5)	0.00
Malignant neoplasm	3 (0.1)	331 (0.0)	0.02	82 (2.6)	5535 (2.5)	0.01
Premature and neonatal	7 (0.1)	684 (0.0)	0.02	3 (0.1)	323 (0.1)	0.02
Miscellaneous, not elsewhere classified	1 (0.0)	67 (0.0)	0.01	7 (0.2)	591 (0.3)	0.01

Abbreviations: MOD, multiple organ dysfunction; NA, not applicable; SMD, standardized mean difference.

^a^ Comparison relevant only to infants; older children had to have at least a 365-day lookback period, so there was no between-cohort difference.

### Outcomes

#### ED Visits

After the index hospitalization, infants in the MOD cohort had more ED visits than the non-MOD cohort in the first 30 days (adjusted IRR, 1.76; 95% CI, 1.56-1.97). The difference persisted in years 1 to 5 (year 1: IRR, 1.86 [95% CI, 1.74-1.99]; year 2: IRR, 1.55 [95% CI, 1.43-1.68]; year 3: IRR, 1.70 [95% CI, 1.52-1.89]; year 4: IRR, 1.77 [95% CI, 1.51-2.06]; year 5: IRR, 1.97 [95% CI, 1.61-2.39]) (Table 2).

**Table 2.** Emergency Department Visits and Rehospitalizations 1 to 5 Years After the Index Hospitalization

Variable	MOD cohort vs non-MOD cohort, IRR (95% CI)				
	Year 1	Year 2	Year 3	Year 4	Year 5
Emergency department visits					
Infants	1.86 (1.74-1.99)	1.55 (1.43-1.68)	1.70 (1.52-1.89)	1.77 (1.51-2.06)	1.97 (1.61-2.39)
Children	1.62 (1.49-1.77)	1.55 (1.36-1.76)	1.70 (1.43-2.03)	1.76 (1.45-2.13)	1.45 (1.15-1.83)
Rehospitalizations					
Infants	10.08 (9.06-11.21)	8.29 (6.99-9.83)	10.23 (7.96-13.16)	11.29 (7.92-16.09)	15.17 (10.17-22.63)
Children	3.71 (3.32-4.14)	2.72 (2.23-3.31)	2.72 (2.11-3.52)	2.53 (1.81-3.53)	2.40 (1.62-3.55)
Infants with MOD^a^	6279	3162	1982	1386	998
Infants without MOD^a^	1 467 235	779 568	509 769	351 473	249 299
Children with MOD^a^	3132	2063	1370	963	652
Children without MOD^a^	219 839	149 002	100 096	70 107	50 176

Abbreviations: IRR, incidence rate ratio; MOD, multiple organ dysfunction.

^a^ Number of patients at the start of each year over time.

For children, there was significant effect modification of ED visits in the first 30 days after the index hospitalization by newly acquired organ-supportive technology during the index hospitalization. As such, ED visits did not differ between the cohorts if there was acquired organ-supportive technology (IRR, 0.97; 95% CI, 0.81-1.17; *P* = .77), but they did differ in the absence of indwelling organ-supportive technology (IRR, 1.71; 95% CI, 1.44-2.03; *P* < .001). Thereafter, the MOD cohort had more ED visits in years 1 to 5 (year 1: IRR, 1.62 [95% CI, 1.49-1.77]; year 2: IRR, 1.55 [95% CI, 1.36-1.76]; year 3: IRR, 1.70 [95% CI, 1.43-2.03]; year 4: IRR, 1.76 [95% CI, 1.45-2.13]; year 5: IRR 1.45 [95% CI, 1.15-1.83]) (Table 2).

#### Rehospitalizations

After the index hospitalization, infants in the MOD cohort had more rehospitalizations than the non-MOD cohort in the first 30 days (IRR, 12.45; 95% CI, 11.40-13.59). The difference persisted in years 1 to 5 (year 1: IRR, 10.08 [95% CI, 9.06-11.21]; year 2: IRR, 8.29 [95% CI, 6.99-9.83]; year 3: IRR, 10.23 [95% CI, 7.96-13.16]; year 4: IRR, 11.29 [95% CI, 7.92-16.09]; year 5: IRR, 15.17 [95% CI, 10.17-22.63]) (Table 2).

For children, rehospitalizations occurred more often in the MOD than non-MOD cohort in the first 30 days (IRR, 4.47; 95% CI, 3.99-5.00). This difference persisted in years 1 to 5 after the index hospitalization (year 1: IRR, 3.71 [95% CI, 3.32-4.14]; year 2: IRR, 2.72 [95% CI, 2.23-3.31]; year 3: IRR, 2.72 [95% CI, 2.11-3.52]; year 4: IRR, 2.53 [95% CI, 1.81-3.53]; year 5: IRR, 2.40 [95% CI, 1.62-3.55]) (Table 2).

#### Health Care Costs

**Figure 2.**
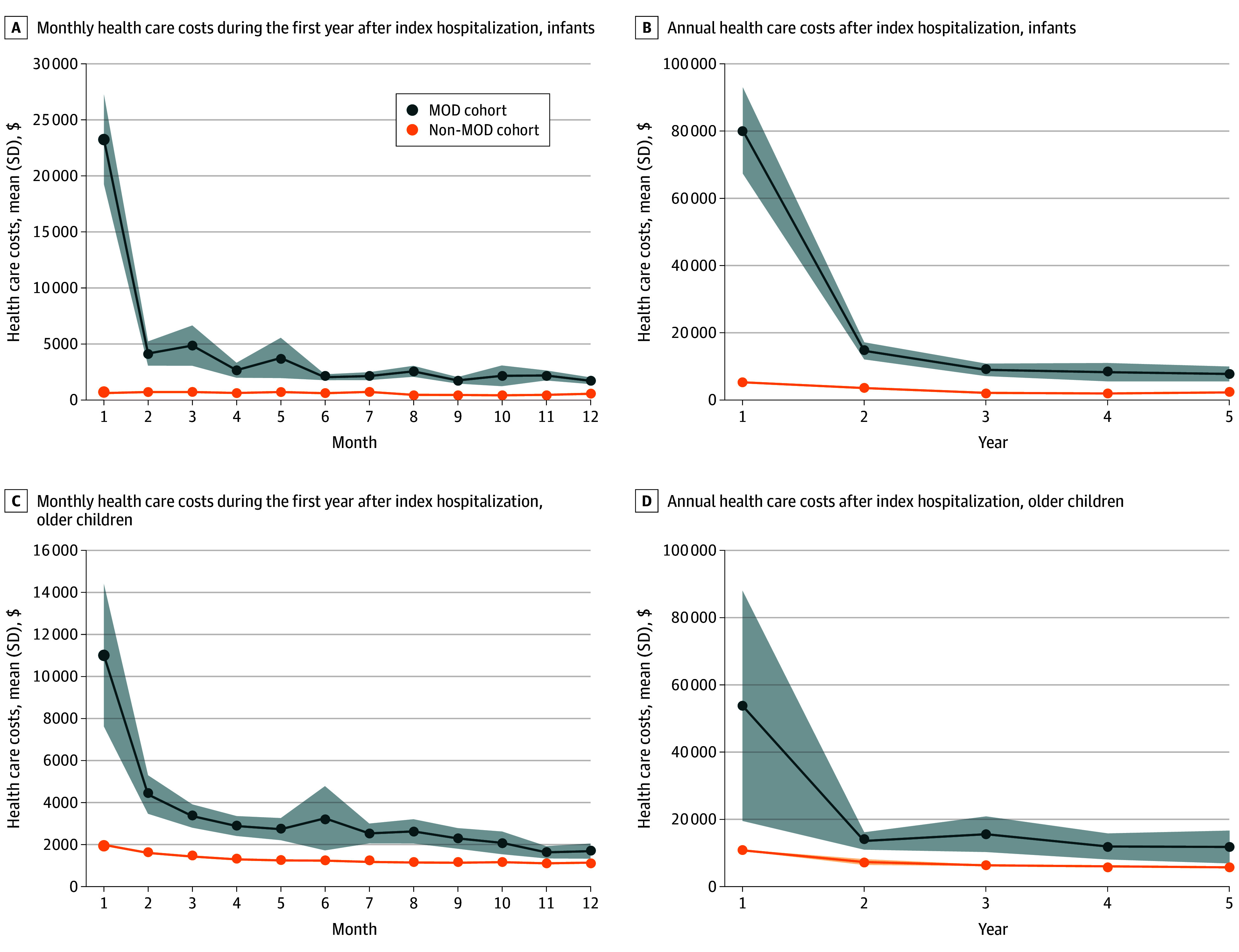
Monthly Health Care Costs During the First Year and Annually for Years 2 Through 5 After the Index Hospitalization MOD indicates multiple organ dysfunction.

Each month in the first year after the index hospitalization, infants and children in the MOD cohort accrued significantly higher health care costs than the non-MOD cohort (infants: mean [SD], $80 133 [$6543] vs $5183 [$19] [*P* < .001]; children: mean [SD], $54 113 [$17 544] vs $10 935 [$95] [*P* < .001]) (Figure 2A and C). This difference persisted throughout the 5 years after the index hospitalization (Figure 2B and D).

### Secondary Outcomes

The MOD cohort had significantly higher use of all secondary measures of health care resources during the entire 5-year follow-up period, particularly during the first year, including any organ-supportive technology (infants, 826 [13.2%] vs 1628 [0.1%] MOD vs non-MOD [*P* < .001]; children, 408 [13.0%] vs 1805 [0.8%] MOD vs non-MOD [*P* < .001]) any rehabilitative therapy services (infants, 1338 [21.3%] vs 30 822 [2.1%] MOD vs non-MOD [*P* < .001]; children, 794 [25.4%] vs 23 878 [10.9%] MOD vs non-MOD [*P* < .001]), and home health services (infants, 598 [9.5%] vs 26 715 [1.8%] MOD vs non-MOD [*P* < .001]; children, 143 [4.6%] vs 1684 [0.8%] MOD vs non-MOD [*P* < .001]). Health care resource use data from 30 days through 5 years after the index hospitalization are provided in eTables 1 to 3 in Supplement 1.

## Discussion

This cohort study highlights the significant association of the development of MOD during a pediatric hospitalization with measures of both short- and long-term clinical and economic burden, including more frequent ED visits, rehospitalizations, and health care costs among the MOD cohort compared with the non-MOD cohort. These findings are an important addition to the growing literature on outcomes after critical pediatric illness and injury, extending the previously reported disparities of higher in-hospital resource burden among patients with MOD compared with those without MOD^30^ to life after hospitalization. This trajectory of high health care resource use and economic burden among survivors of pediatric MOD and their families is a novel finding that suggests adding economic and health care resource use burden to the domains of physical, cognitive, emotional, and social health previously recommended for assessment of survivors of critical illness.^45,46^ With declines in child mortality from critical illness and injury over time,^23^ such longer-term assessments are increasingly important and have been advocated.^46^

The evaluation of health care resource use burden separately among infant and child survivors of MOD also revealed noteworthy within- and between-group similarities and differences. Among infants, there was a consistent difference between cohorts in primary outcomes of ED visits, rehospitalizations, and accrued health care costs at all study time points. Among children, the findings were similar in direction, except for the notable observation of the lack of difference between the MOD and non-MOD cohorts in ED visits within 30 days of discharge from the index hospitalization if organ-supportive medical technology was acquired during the index hospitalization. Several potential device- and patient-related explanations could underlie this important finding. Plausible patient-related explanations include heightened caregiver sensitivity to the maintenance of these indwelling devices, which may prompt ED visits for device evaluation and caregiver reassurance. In addition, the ED visit may enable education and troubleshooting advice regarding care of the organ-supportive devices early in the posthospitalization phase. This speculation is corroborated by recent reports that child survivors of critical illness are increasingly being discharged home with new medical devices and technology,^47^ the care of which may not be fully mastered before discharge from the index hospitalization. Maintenance of such devices may be intimidating at the outset, potentially triggering visits to the ED at the earliest opportunity. Supporting this assertion is a prior study that found a substantial occurrence of ED visits and rehospitalizations within 30 days of gastrostomy,^48^ one of the organ-supportive technologies evaluated in our study.

The starkly higher frequency of ED visits and rehospitalizations among the MOD cohort is concerning for lingering morbidity and medical vulnerability among MOD survivors. Multiple organ dysfunction and the associated need for organ-supportive technology is likely to trigger visits to the health care setting for support of organ function or treatment of acute illness to which the children might be predisposed if organ dysfunction lingers as a chronic morbidity beyond the index hospitalization. A prior study among children who survived hospitalization for sepsis, a leading cause of MOD, found an increase in outpatient health care visits compared with before the hospitalization,^49^ which may indicate ongoing clinical needs potentially from residual morbidity. Future research should elucidate the actual indications for health care encounters after index hospitalizations for MOD.

Accrual of health care costs was resoundingly higher among the MOD cohort up to 5 years after the index hospitalization, likely a reflection of ongoing intensity of health care service needs, costs for the care and maintenance of organ-supportive technology, and the economic impact of rehospitalizations and ED visits. Similar to studies among adult survivors of critical illness,^50,51^ costs were substantially higher in the first year after the index hospitalization than thereafter, suggesting that efforts to mitigate the cost burden for survivors of MOD should be concentrated on the first year after the index hospitalization.

### Limitations

The study findings should be interpreted with consideration of certain limitations. It was not possible to differentiate patient death from disenrollment or loss of insurance coverage. The insurance claims data lacked sufficient clinical information to reliably ascertain the triggers of MOD, limiting the ability to analyze resource use according to triggers of MOD. In addition, the physical and emotional costs to patients and their caregivers that may accrue from rehospitalizations could not be ascertained and warrant future research. Although the cohorts were balanced on observed baseline characteristics, as with any observational study, it is still possible the MOD cohort differed from the non-MOD cohort on some unobserved characteristics that may have differentially influenced the observed outcomes. The absence of an ICU label within the data source precluded the determination of the settings where hospital care was delivered, an important limitation given the knowledge that MOD can arise from ICU treatment.^52^ Severity of MOD during the index hospitalization could not be ascertained definitively, precluding the assessment of any dose-response association between the severity of MOD and postdischarge resource use, costs, and economic burden. Of note, the determination of organ dysfunction, comorbidities, and the use of organ-supportive technology was made via *ICD-9-CM* and *ICD-10-CM* diagnosis and procedure codes and is susceptible to inaccuracies of detection and attribution that may have biased the study findings.

## Conclusions

This cohort study serves as a pivotal first step toward enhanced knowledge of the posthospitalization epidemiology of pediatric MOD, particularly the resultant long-term health care resource use and costs of care. We have uncovered that children who survive hospitalization with MOD are considerably more susceptible to a posthospitalization trajectory of ED visits and rehospitalizations compared with children without MOD. Efforts to support survivors of MOD should incorporate the highlighted economic burden into existing or new paradigms of posthospitalization follow-up and care. Urgent efforts are needed to prevent MOD during the initial hospitalization, with an emphasis on identifying high-risk subgroups that could be targeted for close clinical follow-up and interventions to prevent MOD or aggressively mitigate its outcomes. Assessment of child and family health and well-being after critical illness with MOD should incorporate health care resource use and economic burden. The study findings are likely generalizable to hospitals that deliver care to children with severe illness or injury.

## Notes

Supplement 1. **eTable 1.** Use of Organ-Supportive Technology, Rehabilitation, and Home Health Services at 30 Days After Discharge From Index Hospitalization **eTable 2.** Use of Organ-Supportive Technology, Rehabilitation, and Home Health Services at 1 Year After Discharge From Index Hospitalization **eTable 3.** Use of Organ-Supportive Technology, Rehabilitation, and Home Health Services at 2 to 5 Years After Discharge From Index Hospitalization **eAppendix 1.** Organ Dysfunction Codes **eAppendix 2.** Categories of Comorbidities and the Corresponding *ICD-9* and *ICD-10* Diagnosis and Procedure Codes

Supplement 2.
Data Sharing Statement
