# Supplementary material for: Health Care Resource Use and Costs After Hospitalization With Multiple Organ Dysfunction in Children
Source: JAMA Netw Open. 2025 Jan 29;8(1):e2456246. doi: 10.1001/jamanetworkopen.2024.56246 (PMC11780478; doi:10.1001/jamanetworkopen.2024.56246)
Supplement: Supplement 1. — eTable 1. Use of Organ-Supportive Technology, Rehabilitation, and Home Health Services at 30 Days After Discharge From Index Hospitalization eTable 2. Use of Organ-Supportive Technology, Rehabilitation, and Home Health Services at 1 Year After Discharge From Index Hospitalization eTable 3. Use of Organ-Supportive Technology, Rehabilitation, and Home Health Services at 2 to 5 Years After Discharge From Index Hospitalization eAppendix 1. Organ Dysfunction Codes eAppendix 2. Categories of Comorbidities and the Corresponding ICD-9 and ICD-10 Diagnosis and Procedure Codes [file jamanetwopen-e2456246-s001.pdf]

Supplemental Online Content

Odetola FO, Lin P, Ye W, Dombkowski KJ, Linden A. Health care resource use and costs after hospitalization for multiple organ dysfunction in children. *JAMA Netw Open*. 2025;8(1):e2456246. doi:10.1001/jamanetworkopen.2024.56246

eTable 1. Use of Organ-Supportive Technology, Rehabilitation, and Home Health Services at 30 Days After Discharge From Index Hospitalization

eTable 2. Use of Organ-Supportive Technology, Rehabilitation, and Home Health Services at 1 Year After Discharge From Index Hospitalization

eTable 3. Use of Organ-Supportive Technology, Rehabilitation, and Home Health Services at 2 to 5 Years After Discharge From Index Hospitalization

eAppendix 1. Organ Dysfunction Codes

eAppendix 2. Categories of Comorbidities and the Corresponding *ICD-9* and *ICD-10* Diagnosis and Procedure Codes

This supplemental material has been provided by the authors to give readers additional information about their work.

© 2025 Odetola F et al. *JAMA Network Open.*

eTable 1. Use of Organ-Supportive Technology, Rehabilitation, and Home Health Services at 30 Days After Discharge From Index Hospitalization

	Infants			Older children		
**Variable**	**MOD cohort (n=6,279)**	**Non-MOD cohort (n=1,467,235)**	**p**	**MOD cohort (n=3,132)**	**Non-MOD cohort (n=219,839)**	**p**
**Technology**						
Any	521 (8·3)	768 (0·1)	<·01	279 (8·9)	897 (0·4)	<·01
Dialysis	34 (0·5)	8 (<0·1)	<·01	35 (1·1)	7 (<0·1)	<·01
Tracheostomy	52 (0·8)	9 (<0·1)	<·01	83 (2·7)	39 (<0·1)	<·01
Gastrostomy	201 (3·2)	178 (<0·1)	<·01	146 (4·7)	251 (0·1)	<·01
Central venous access device	278 (4·4)	465 (<0·1)	<·01	95 (3·0)	585 (0·3)	<·01
Nasal/oral-enteral feeding tube	89 (1·4)	127 (<0·1)	<·01	26 (0·8)	61 (<0·1)	<·01
Cerebral ventricular shunt device	22 (0·4)	31 (<0·1)	<·01	8 (0·3)	13 (<0·1)	0·02
**Rehabilitation**						
Any	245 (3·9)	2,551 (0·2)	<·01	435 (13·9)	9,950 (4·5)	<·01
Physical Therapy	176 (2·8)	1,744 (0·1)	<·01	391 (12·5)	8,663 (3·9)	<·01
Occupational Therapy	122 (1·9)	830 (0·1)	<·01	218 (7·0)	1,029 (0·5)	<·01
Speech Therapy	41 (0·7)	339 (<0·1)	<·01	181 (5·8)	1,190 (0·5)	<·01
Music Therapy	—	—	—	3 (0·1)	—	<·01
**Home health services**	433 (6·9)	26,386 (1·8)	<·01	94 (3·0)	1462 (0·7)	<·01

discharge from index hospitalization

Data are number (%) unless stated otherwise.

© 2025 Odetola F et al. *JAMA Network Open.*

eTable 2. Use of Organ-Supportive Technology, Rehabilitation, and Home Health Services at 1 Year After Discharge From Index Hospitalization

	Infants			Older children		
**Variable**	**MOD cohort (n = 6,279)**	**Non-MOD cohort (n=1,467,235)**	**p**	**MOD cohort (n = 3,132)**	**Non-MOD cohort (n=219,839)**	**p**
**Technology**						
Any	826 (13·2)	1628 (0·1)	<·01	408 (13·0)	1805 (0·8)	<·01
Dialysis	44 (0·7)	32 (<0·1)	<·01	48 (1·5)	23 (<0·1)	<·01
Tracheostomy	92 (1·5)	21 (<0·1)	<·01	107 (3·4)	62 (<0·1)	<·01
Gastrostomy	405 (6·5)	371 (<0·1)	<·01	213 (6·8)	600 (0·3)	<·01
Central venous access device	496 (7·9)	990 (0·1)	<·01	208 (6·6)	1102 (0·5)	<·01
Nasal/oral-enteral feeding tube	181 (2·9)	282 (<0·1)	<·01	67 (2·1)	156 (0·1)	<·01
Cerebral ventricular shunt device	50 (0·8)	82 (<0·1)	<·01	20 (0·6)	35 (<0·1)	<·01
**Rehabilitation**						
Any	1,338 (21·3)	30,822 (2·1)	<·01	794 (25·4)	23,878 (10·9)	<·01
Physical Therapy	1,108 (17·6)	27,110 (1·8)	<·01	719 (23·0)	21,056 (9·6)	<·01
Occupational Therapy	672 (10·7)	5,771 (0·4)	<·01	371 (11·8)	3,040 (1·4)	<·01
Speech Therapy	322 (5·1)	2,531 (0·2)	<·01	338 (10·8)	3,047 (1·4)	<·01
Music Therapy	1 (0·0)	1 (0·0)	0·35	4 (0·1)	—	<·01
**Home health services**	598 (9·5)	26715 (1·8)	<·01	143 (4·6)	1684 (0·8)	<·01

Data are number (%) unless stated otherwise.

© 2025 Odetola F et al. *JAMA Network Open.*

eTable 3. Use of Organ-Supportive Technology, Rehabilitation, and Home Health Services at 2 to 5 Years After Discharge From Index Hospitalization

Year 2	Infants			Older children		
**Variable**	**MOD cohort (n = 3,162)**	**Non-MOD cohort (n=779,568)**	**p**	**MOD cohort (n = 2,063)**	**Non-MOD cohort (n=149,002)**	**p**
Technology	219 (6·9)	354 (<0·1)	<·01	135 (6·5)	538 (0·4)	<·01
Rehabilitation	635 (20·1)	16,600 (2·1)	<·01	251 (12·2)	8,574 (5·8)	<·01
Home health services	78 (2·5%)	137 (<0·1)	<·01	26 (1·3%)	159 (0·1%)	<·01
**Year 3**	**Infants**			**Older children**		
**Variable**	**MOD cohort (n = 1,982)**	**Non-MOD cohort (n=509,769)**	**p**	**MOD cohort (n = 1,370)**	**Non-MOD cohort (n=100,096)**	**p**
Technology	127 (6·4)	148 (<0·1)	<·01	83 (6·1)	272 (0·3)	<·01
Rehabilitation	373 (18·8)	16,065 (3·2)	<·01	154 (11·2)	5,185 (5·2)	<·01
Home health services	25 (1·3)	67 (<0·1)	<·01	17 (1·2)	93 (0·1)	<·01
**Year 4**	**Infants**			**Older children**		
**Variable**	**MOD cohort (n = 1,386)**	**Non-MOD cohort (n=351,473)**	**p**	**MOD cohort (n = 963)**	**Non-MOD cohort (n=70,107)**	**p**
Technology	83 (6·0)	90 (<0·1)	<·01	50 (5·2)	153 (0·2)	<·01
Rehabilitation	185 (13·3)	8,655 (2·5)	<·01	97 (10·1)	3,521 (5·0)	<·01
Home health services	20 (1·4%)	17 (0·0%)	<·01	11 (1·1%)	54 (0·1%)	0·02
**Year 5**	**Infants**			**Older children**		
**Variable**	**MOD cohort (n = 998)**	**Non-MOD cohort (n=249,299)**	**p**	**MOD cohort (n = 652)**	**Non-MOD cohort (n=50,176)**	**p**
Technology	57 (5·7)	55 (<0·1)	<·01	27 (4·1)	122 (0·2)	<·01
Rehabilitation	125 (12·5)	6,837 (2·7)	<·01	65 (10·0)	2,294 (4·6)	<·01
Home health services	13 (1·3%)	18 (0·0%)	<0.01	4 (0·6%)	29 (0·1%)	0·17

Data are number (%) unless stated otherwise.

© 2025 Odetola F et al. *JAMA Network Open.*

eAppendix 1. Organ Dysfunction Codes

Organ System		ICD-9	ICD-10
**Respiratory**			
	Acute respiratory failure	51881	J960, J9600, 9601, J9602, J969, J9690, J9691, J9692
	Acute respiratory distress, Insufficiency	51882	J80, R0603
	Acute-on-chronic respiratory failure	51884	J962, J9620, J9621, J9622
	Pulmonary insufficiency following trauma and surgery	5185, 51851 51852, 51853	J951, J952, J953, J9582, J95821, J95822, J960, J9600, J9601 J9602, J962, J9620, J9621, J9622
	Respiratory arrest, cardiorespiratory failure	7991	R092
	Other respiratory problems after birth	77089	P228, P229, P2889
	Mechanical ventilation (procedure codes)	967, 9670, 9671 9672	5A1935Z, 5A1945Z, 5A1955Z
**Cardiovascular**			
	Cardiac arrest	4275	I46, I462, I468, I469
	Hypotension	458, 4580, 4581 4582, 45821, 45829, 4588 4589	I95, I950, I951, I952, I953, I958, I9581, I9589, I959
	Shock unspecified, Failure of peripheral circulation	78550	R579
	Other shock, Septic	78559	R571, R578
**Neurologic**			
	Transient organic psychosis	293, 2930, 2931 2938, 29381 29382, 29383 29384, 29389 2939	F05, F06, F060, F061, F062, F063, F0630, F0631 F0632, F0633, F0634, F064, F068, F53, F530, F531
	Anoxic brain injury	3481	G931
	Acute encephalopathy	3483, 34830, 34831, 34839	G934, G9340, G9341, G9349, I6783
	Critical illness myopathy	35981	G7281
	Hepatic encephalopathy	5722	K7041, K7111, K7211, K7290, K7291
	Coma	78001	R402, R4020, R4021, R40211, R402110, R402111 R402112, R402113, R402114, R40212, R402120 R402121, R402122, R402123, R402124,

© 2025 Odetola F et al. *JAMA Network Open.*

			R40213<br>R402130, R402131, R402132, R402133, R402134<br>R40214, R402140, R402141, R402142, R402143<br>R402144, R4022, R40221, R402210, R402211<br>R402212, R402213, R402214, R40222, R402220<br>R402221, R402222, R402223, R402224, R40223<br>R402230, R402231, R402232, R402233, R402234<br>R40224, R402240, R402241, R402242, R402243<br>R402244, R40225, R402250, R402251, R402252<br>R402253, R402254, R4023, R40231, R402310<br>R402311, R402312, R402313, R402314, R40232<br>R402320, R402321, R402322, R402323, R402324<br>R40233, R402330, R402331, R402332, R402333<br>R402334, R40234, R402340, R402341, R402342<br>R402343, R402344, R40235, R402350, R402351<br>R402352, R402353, R402354, R40236, R402360<br>R402361, R402362, R402363, R402364, R4024<br>R40241, R402410, R402411, R402412, R402413<br>R402414, R40242, R402420, R402421, R402422<br>R402423, R402424, R40243, R402430, R402431<br>R402432, R402433, R402434, R40244, R402440<br>R402441, R402442, R402443, R402444, E035
	Altered consciousness, Unspecified	78009	R400, R401
**Hepatic**			
	Acute and subacute necrosis of liver	570	K720, K7200, K7201, K762
	Hepatic infarction	5734	K763
**Hematologic**			
	Defibrination syndrome, disseminated intravascular coagulation	2866	D65
	Other and unspecified	2869	D688, D689

© 2025 Odetola F et al. *JAMA Network Open.*

	coagulation defects		
	Secondary thrombocytopenia	2874, 28741 28749	D695, D6951, D6959
	Thrombocytopenia, Unspecified	2875	D696
**Renal**			
	Acute renal failure	584, 5845 5846, 5847 5848, 5849	N17, N170, N171, N172, N178, N179
	Renal failure, Unspecified	586	N19
	Oliguria, Anuria	7885	R34
	Nephritis and nephropathy, not specified as acute or chronic, with lesion of renal cortical necrosis	5836	N171
	Nephritis and nephropathy, not specified as acute or chronic, with lesion of renal medullary necrosis	5837	N172

ICD = International Classification of Diseases

© 2025 Odetola F et al. *JAMA Network Open.*

eAppendix 2. Categories of Comorbidities and the Corresponding *ICD-9* and *ICD-10* Diagnosis and Procedure Codes*

Categories	Subcategories	ICD-9	ICD-10
**Neurologic and Neuromuscular**	Brain and spinal cord malformations	740.0-742.9	Q00-Q07, G90.1
	Mental retardation	318.0-318.2	F71-F73
	CNS degeneration and diseases	330.0-330.9, 334, 335.0-335.9, 331.1, 331.11, 331.19, 331.4, 331.8, 331.89, 331.9, 333.2, 336.1, 336.8, 337.9, 759.5	E75.0, E75.1, E75.2, E75.4, F84.2, G11.1-G11.4, G11.8, G11.9, G12.0- G12.2, G12.8, G12.9, G31.01, G31.09, G31.8, G31.89, G32.89, G93.8, G93.9, G94, G91.1, G31.9, G25.3, G95.19, G95.89, G90.9, Q85.1
	Infantile cerebral palsy	343.0-343.9	G80
	Epilepsy	345.01, 345.11, 345.3, 345.41, 345.61, 345.71, 345.81, 345.91	G40.311, G40.301, G40.211, G40.219, G40.411, G40.419, G40.111, G40.119, G40.804, G40.911, G40.919
	Other disorders of CNS	341.8, 342.90, 344.0, 344.81, 344.9, 348.1, 348.4, 780.03, 01.52, 01.53	G37.1, G37.2, G37.8, G81.90, G82.90, G82.50-G82.54, G83.5, G83.9, G93.1, G93.5, R40.3, 0016070, 0016071, 0016072, 0016073, 0016074, 0016075, 0016076, 0016077, 0016078, 001607B, 0016370, 0016371, 0016372, 0016373, 0016374, 0016375, 0016376, 0016377, 0016378, 001637B, 001U074, 001U076, 001U077, 001U079, 001U374, 001U376, 001U377, 001U379, 00B70ZZ, 00B73ZZ, 00B74ZZ, 00T70ZZ, 00T73ZZ, 00T74ZZ
	Occlusion of cerebral arteries	434.01, 434.91	I63.30, I63.50
	Muscular dystrophies and myopathies	359.0-359.3	G71, G72
	Movement diseases	332.0, 332.1, 333.0, 333.2,33.4, 333.5, 333.7, 333.9	G10, G20, G21.0, G21.11, G21.19, G21.8, G23.0-G23.2, G23.8, G24.02, G24.8, G25.3-G25.5, G25.81-G25.83, G25.89, G25.9, G80.3
	Devices	996.2, 996.63, V45.2, V53.01, V53.02, 02.2, 02.21, 02.22, 02.3, 02.31, 02.32, 02.33, 02.34, 02.35, 02.39, 02.4, 02.41, 02.42, 02.93, 03.7, 03.71, 03.72. 03.79, 03.93, 03.97, 04.92	T85.09XA, T85.190A, T85.192A, T85.199A, T85.79XA, Z98.2, Z45.41, Z45.42, 00160J0, 00160J1, 00160J2, 00160J3, 00160J4, 00160J5, 00160J6, 00160J7, 00160J8, 00160JB, 00160K0, 00160K1, 00160K2, 00160K3, 00160K4, 00160K5, 00160K6, 00160K7, 00160K8, 00160KB, 00163J0, 00163J1,

© 2025 Odetola F et al. *JAMA Network Open.*

Categories	Subcategories	ICD-9	ICD-10
			00163J2, 00163J3, 00163J4, 00163J5, 00163J6, 00163J7, 00163J8, 00163JB, 00163K0, 00163K1, 00163K2, 00163K3, 00163K4, 00163K5, 00163K6, 00163K7, 00163K8, 00163KB, 001U0J4, 001U0J6, 001U0J7, 001U0J9, 001U0K4, 001U0K6, 001U0K7, 001U0K9, 001U3J4, 001U3J6, 001U3J7, 001U3J9, 001U3K4, 001U3K6, 001U3K7, 001U3K9, 009600Z, 009630Z, 009640Z, 00H00MZ, 00H03MZ, 00H04MZ, 00H60MZ, 00H63MZ, 00H64MZ, 00HE0MZ, 00HE3MZ, 00HE4MZ, 00HU0MZ, 00HU3MZ, 00HU4MZ, 00HV0MZ, 00HV3MZ, 00HV4MZ, 00W60JZ, 00W63JZ, 00W64JZ, 00WU0JZ, 00WU3JZ, 00WU4JZ, 01HY0MZ, 01HY3MZ, 01HY4MZ, 0DH60MZ, 0DH63MZ, 0DH64MZ, 0W110J9, 0W110JB, 0W110JG, 0W110JJ, 3E1Q38X, 3E1Q38Z
	Transplantation	N/A	N/A
**Cardiovascular**	Heart and great vessel malformations	745.0-745.3, 745.60-745.69, 746, 747.1-747.49, 747.81, 747.89, 35.8, 35.81, 35.82, 35.83, 35.84	Q20, Q21.2-Q24, Q25.1-Q26, Q28.2, Q28.3, Q28.9, 02170ZP, 02170ZQ, 02170ZR, 02BK0ZZ, 02LR0ZT, 02LS0ZZ, 02LT0ZZ, 02NH0ZZ, 02RK0JZ, 02RL0JZ, 02RM0JZ, 02RP0JZ, 02RQ07Z, 02RQ0JZ, 02RR07Z, 02RR0JZ, 02SP0ZZ, 02SW0ZZ, 02U70JZ, 02UA0JZ, 02UA3JZ, 02UA4JZ, 02VR0ZT, 02WA0JZ
	Endocardium diseases	424.0, 424.2, 424.3	I34.0, I34.8, I36.0, I36.8, I37.0, I37.8
	Cardiomyopathies	425.0-425.4, 425.8, 429.1	I42, I43, I51.5
	Conduction disorder	426.0-427.4	I44, I45, I47, I48, I49.0
	Dysrhythmias	427.6-427.9	I49.1-I49.5, I49.8, I49.9, R00.1
	Other	416.1, 416.8, 416.9, 428.0, 429.3, 428.83, 433.11, V45.81	I27.0, I27.1, I27.2, I27.81, I27.89, I27.9, I50.9, I51.7, I51.81, I63.139, I63.239, Z95.1
	Devices	996.0, 996.1, 996.61, 996.62, V43.3, V45.0, V53.31, V53.32, V53.39, 00.50, 00.51, 00.53, 00.54, 00.55, 00.57, 17.51,	T82.519A, T82.529A, T82.539A, T82.599A, T82.110A, T82.111A, T82.120A, T82.121A, T82.190A, T82.191A, T82.01XA, T82.02XA, T82.03XA, T82.09XA, T82.211A,
		17.52, 37.41, 37.52, 37.53, 37.54, 37.55,	T82.212A, T82.213A, T82.218A, T82.221A, T82.222A,

© 2025 Odetola F et al. *JAMA Network Open.*

Categories	Subcategories	ICD-9	ICD-10
		37.6, 37.60, 37.61, 37.63, 37.65, 37.66, 37.67, 37.68, 37.7, 37.71, 37.72, 37.74, 37.76, 37.79, 37.8, 37.80, 37.81, 37.82, 37.83, 37.85, 37.86, 37.87, 37.89, 37.94, 37.95, 37.96, 37.97, 37.98, 39.81, 39.82, 39.83, 39.84, 39.85, 89.46, 89.47, 89.48, 89.49	T82.223A, T82.228A, T82.518A, T82.528A, T82.538A, T82.598A, T82.6XXA, T82.7XXA, Z95.0, Z95.2, Z95.3, Z95.810-Z95.812, Z95.818, Z45.010, Z45.018, Z45.02, Z45.09, Z95.9, 02H40JZ, 02H40KZ, 02H43JZ, 02H44JZ, 02H44KZ, 02H60JZ, 02H60KZ, 02H63JZ, 02H63KZ, 02H63MZ, 02H64JZ, 02H64KZ, 02H70KZ, 02H73JZ, 02H73KZ, 02H73MZ, 02H74KZ, 02HA0QZ, 02HA0RS, 02HA0RZ, 02HA3QZ, 02HA3RS, 02HA4QZ, 02HA4RS, 02HK0JZ, 02HK0KZ, 02HK3JZ, 02HK3KZ, 02HK3MZ, 02HK4JZ, 02HK4KZ, 02HL0JZ, 02HL0KZ, 02HL0MZ, 02HL3JZ, 02HL3KZ, 02HL3MZ, 02HL4JZ, 02HL4KZ, 02HL4MZ, 02HN0JZ, 02HN0KZ, 02HN0MZ, 02HN3JZ, 02HN3KZ, 02HN3MZ, 02HN4JZ, 02HN4KZ, 02HN4MZ, 02WA0QZ, 02WA0RZ, 02WA3QZ, 02WA3RZ, 02WA4QZ, 02WA4RZ, 03HK0MZ, 03HK3MZ, 03HK4MZ, 03HL0MZ, 03HL3MZ, 03HL4MZ, 03WY0MZ, 03WY3MZ, 03WY4MZ, 0JH600Z, 0JH605Z, 0JH606Z, 0JH607Z, 0JH608Z, 0JH609Z, 0JH60AZ, 0JH60MZ, 0JH60PZ, 0JH630Z, 0JH635Z, 0JH636Z, 0JH637Z, 0JH638Z, 0JH639Z, 0JH63AZ, 0JH63MZ, 0JH63PZ, 0JH70MZ, 0JH73MZ, 0JH800Z, 0JH805Z, 0JH806Z, 0JH807Z, 0JH808Z, 0JH809Z, 0JH80AZ, 0JH80MZ, 0JH80PZ, 0JH830Z, 0JH835Z, 0JH836Z, 0JH837Z, 0JH838Z, 0JH839Z, 0JH83AZ, 0JH83MZ, 0JH83PZ, 0JWT0MZ, 0JWT0PZ, 0JWT3MZ, 0JWT3PZ, 0JWTXMZ, 4B02XSZ, 4B02XTZ, 5A02110, 5A02116, 5A0211D, 5A02210, 5A02216, 5A0221D
	Transplantation	996.83, V42.1, V42.2, V43.2, 37.5, 37.51	T86.20-T86.22, Z94.1, 02YA0Z0, 02YA0Z1, 02YA0Z2
**Respiratory**	Respiratory malformations	748.0-748.9	Q30-Q34, P280
	Chronic respiratory diseases	327.25, 416.2, 516.3, 516.31, 518.84, 770.4, V45.76	G47.35, I27.82, I43, J84.112, J96.20, Z90.2
	Cystic fibrosis	277.0	E84

© 2025 Odetola F et al. *JAMA Network Open.*

Categories	Subcategories	ICD-9	ICD-10
	Other	30.3, 30.4, 32.4, 32.41, 32.49, 32.5, 32.50, 32.59	0B110Z4, 0B113Z4, 0B114Z4, 0BTC0ZZ, 0BTC4ZZ, 0BTD0ZZ, 0BTD4ZZ, 0BTF0ZZ, 0BTF4ZZ, 0BTG0ZZ, 0BTG4ZZ, 0BTJ0ZZ, 0BTJ4ZZ, 0BTK0ZZ, 0BTK4ZZ, 0BTL0ZZ, 0BTL4ZZ, 0BTM0ZZ, 0BTM4ZZ, 0CTS0ZZ, 0CTS4ZZ, 0CTS7ZZ, 0CTS8ZZ
	Devices	519.0, V44.0, V55.0, V46.0, V46.1, 31.2, 31.21, 31.29, 31.41, 31.74, 33.21, 34.85, 96.55, 97.23	J95.00-J95.04, J95.09, Z43.0, Z93.0, Z99.0, J95.850, Z99.11, Z99.12, 0B110F4, 0B113F4, 0B114F4, 0B21XFZ, 0BHR0MZ, 0BHR3MZ, 0BHR4MZ, 0BHS0MZ, 0BHS3MZ, 0BHS4MZ, 0BW10FZ, 0BW13FZ, 0BW14FZ, 0JH604Z, 0JH634Z, 0JH804Z, 0JH834Z, 0WQ6XZ2, 3E1F78Z
	Transplantation	996.84, V42.6, 33.5, 33.50, 33.51, 33.52, 33.6	T86.810, T86.811, T86.819, Z94.2, 0BYC0Z0, 0BYC0Z1, 0BYC0Z2, 0BYD0Z0, 0BYD0Z1, 0BYD0Z2, 0BYF0Z0, 0BYF0Z1, 0BYF0Z2, 0BYG0Z0, 0BYG0Z1, 0BYG0Z2, 0BYH0Z0, 0BYH0Z1, 0BYH0Z2, 0BYJ0Z0, 0BYJ0Z1, 0BYJ0Z2, 0BYK0Z0, 0BYK0Z1, 0BYK0Z2, 0BYL0Z0, 0BYL0Z1, 0BYL0Z2, 0BYM0Z0, 0BYM0Z1, 0BYM0Z2
**Renal and Urologic**	Congenital anomalies	753.0-753.9	Q60-Q64
	Chronic renal failure	585	N18
	Other	V45.73, V45.74, 55.5, 55.51, 55.52, 55.53, 55.54, 56.4, 56.41, 56.42, 56.7, 56.71, 56.79, 57.7, 57.71, 57.79	Z90.5, Z90.6, 0T160Z8, 0T160ZA, 0T164Z8, 0T164ZA, 0T170Z8, 0T170ZA, 0T174Z8, 0T174ZA, 0T180Z8, 0T180ZA, 0T184Z8, 0T184ZA, 0TB60ZZ, 0TB63ZZ, 0TB64ZZ, 0TB67ZZ, 0TB68ZZ, 0TB70ZZ, 0TB73ZZ, 0TB74ZZ, 0TB77ZZ, 0TB78ZZ, 0TT00ZZ, 0TT04ZZ, 0TT10ZZ, 0TT14ZZ, 0TT20ZZ, 0TT24ZZ, 0TT60ZZ, 0TT64ZZ, 0TT67ZZ, 0TT68ZZ, 0TT70ZZ, 0TT74ZZ, 0TT77ZZ, 0TT78ZZ, 0TTB0ZZ, 0TTB4ZZ, 0TTB7ZZ, 0TTB8ZZ, 0TTD0ZZ, 0TTD4ZZ, 0TTD7ZZ, 0TTD8ZZ
	Chronic bladder diseases	344.61, 596.4, 596.53, 596.54	G83.4, N31.2, N31.9

© 2025 Odetola F et al. *JAMA Network Open.*

Categories	Subcategories	ICD-9	ICD-10
	Devices	996.68, V44.5, V44.6, V45.1, V53.6, V55.5, V55.6, 38.95, 39.27, 39.42, 39.93, 39.94, 39.95, 54.98, 55.02, 55.03, 55.04, 55.12, 55.93, 55.94, 55.97, 56.5, 56.51, 56.52, 56.6, 56.61, 56.62, 56.72, 56.73, 56.74, 56.75, 57.2, 57.21, 57.22, 59.93, 59.94, 86.07, 96.45, 96.46, 96.47	T85.71XA, Z93.50-Z93.52, Z93.59, Z93.6, Z91.15, Z99.2, Z43.5, Z43.6, Z46.6, 031209D, 031209F, 03120AD, 03120AF, 03120JD, 03120JF, 03120KD, 03120KF, 03120ZD, 03120ZF, 031309D, 031309F, 03130AD, 03130AF, 03130JD, 03130JF, 03130KD, 03130KF, 03130ZD, 03130ZF, 031409D, 031409F, 03140AD, 03140AF, 03140JD, 03140JF, 03140KD, 03140KF, 03140ZD, 03140ZF, 031509D, 031509F, 03150AD, 03150AF, 03150JD, 03150JF, 03150KD, 03150KF, 03150ZD, 03150ZF, 031609D, 031609F, 03160AD, 03160AF, 03160JD, 03160JF, 03160KD, 03160KF, 03160ZD, 03160ZF, 031709D, 031709F, 03170AD, 03170AF, 03170JD, 03170JF, 03170KD, 03170KF, 03170ZD, 03170ZF, 031809D, 031809F, 03180AD, 03180AF, 03180JD, 03180JF, 03180KD, 03180KF, 03180ZD, 03180ZF, 031909F, 03190AF, 03190JF, 03190KF, 03190ZF, 031A09F, 031A0AF, 031A0JF, 031A0KF, 031A0ZF, 031B09F, 031B0AF, 031B0JF, 031B0KF, 031B0ZF, 031C09F, 031C0AF, 031C0JF, 031C0KF, 031C0ZF, 03WY0JZ, 03WY3JZ, 03WY4JZ, 03WYXJZ, 05HY33Z, 06HY33Z, 0JH60WZ, 0JH60XZ, 0JH63WZ, 0JH63XZ, 0JH80WZ, 0JH80XZ, 0JH83WZ, 0JH83XZ, 0JHD0WZ, 0JHD0XZ, 0JHD3WZ, 0JHD3XZ, 0JHF0WZ, 0JHF0XZ, 0JHF3WZ, 0JHF3XZ, 0JHL0WZ, 0JHL0XZ, 0JHL3WZ, 0JHL3XZ, 0JHM0WZ, 0JHM0XZ, 0JHM3WZ, 0JHM3XZ, 0T130ZB, 0T134ZB, 0T140ZB, 0T144ZB, 0T16079, 0T1607C, 0T1607D, 0T160J9, 0T160JC, 0T160JD, 0T160K9, 0T160KC, 0T160KD, 0T160Z9, 0T160ZC, 0T160ZD, 0T163JD, 0T16479, 0T1647C, 0T1647D, 0T164J9, 0T164JC, 0T164JD, 0T164K9, 0T164KC, 0T164KD, 0T164Z9, 0T164ZC, 0T164ZD, 0T17079, 0T1707C, 0T1707D, 0T170J9, 0T170JC, 0T170JD, 0T170K9, 0T170KC, 0T170KD, 0T170Z9, 0T170ZC, 0T170ZD, 0T173JD, 0T17479, 0T1747C, 0T1747D, 0T174J9, 0T174JC, 0T174JD, 0T174K9, 0T174KC, 0T174KD, 0T174Z9, 0T174ZC, 0T174ZD, 0T18079, 0T1807C, 0T1807D, 0T180J9,

© 2025 Odetola F et al. *JAMA Network Open.*

Categories	Subcategories	ICD-9	ICD-10
			0T180JC, 0T180JD, 0T180K9, 0T180KC, 0T180KD, 0T180Z9, 0T180ZC, 0T180ZD, 0T183JD, 0T18479, 0T1847C, 0T1847D, 0T184J9, 0T184JC, 0T184JD, 0T184K9, 0T184KC, 0T184KD, 0T184Z9, 0T184ZC, 0T184ZD, 0T1B0ZD, 0T1B4ZD, 0T25X0Z, 0T29X0Z, 0T29XYZ, 0T2BX0Z, 0T9000Z, 0T9030Z, 0T9040Z, 0T9070Z, 0T9080Z, 0T9100Z, 0T9130Z, 0T9140Z, 0T9170Z, 0T9180Z, 0T9370Z, 0T9380Z, 0T9470Z, 0T9480Z, 0TQ67ZZ, 0TQ77ZZ, 3E1K38Z, 3E1M39Z, 5A1D60Z
	Transplantation	996.81, V42.0, 55.6, 55.61, 55.69	T86.10-T86.12, Z94.0, 0TY00Z0, 0TY00Z1, 0TY00Z2, 0TY10Z0, 0TY10Z1, 0TY10Z2
**Gastrointestinal**	Congenital anomalies	750.3, 751.1-751.9	Q39.0-Q39.4, Q41-Q45
	Chronic liver disease and cirrhosis	571.4-571.9	K73, K74, K75.4, K760-K763, K765, K768
	Inflammatory bowel diseases	555.0-556.9	K50, K51
	Other	453.0, 557.1, 560.2, 564.7, V45.3, V45.72, V45.75, 25.3, 25.4, 42.42, 43.9, 43.91, 43.99, 45.63, 45.8, 45.81, 45.82, 45.83, 50.4, 52.6, 52.7, 54.71	I82.0, K55.1, K56.2, K59.3, Z98.0, Z90.3, Z90.49, 0CT70ZZ, 0CT7XZZ, 0D13079, 0D1307A, 0D1307B, 0D1607A, 0D160ZA, 0DT50ZZ, 0DT54ZZ, 0DT57ZZ, 0DT58ZZ, 0DT60ZZ, 0DT64ZZ, 0DT67ZZ, 0DT68ZZ, 0DT80ZZ, 0DT84ZZ, 0DT87ZZ, 0DT88ZZ, 0DT90ZZ, 0DT94ZZ, 0DT97ZZ, 0DT98ZZ, 0DTE0ZZ, 0DTE4ZZ, 0DTE7ZZ, 0DTE8ZZ, 0FT00ZZ, 0FT04ZZ, 0FTG0ZZ, 0FTG4ZZ

© 2025 Odetola F et al. *JAMA Network Open.*

Categories	Subcategories	ICD-9	ICD-10
	Devices	536.4, V44.1-V44.4, V53.50, V53.51, V53.59, V55.1-V55.4, 42.1, 42.10, 42.11, 42.81, 43.1, 43.11, 43.19, 44.12, 44.3, 44.32, 44.38, 44.39, 46.1, 46.13, 46.2, 46.22, 46.23, 46.3, 46.32, 46.4, 46.40, 46.41, 46.43, 96.24, 96.36, 97.02	K94.20, K94.22, K94.23, K94.29, Z93.1-Z93.4, Z43.1-Z43.4, Z46.51, Z46.59, 0D11074, 0D110J4, 0D110K4, 0D110Z4, 0D113J4, 0D11474, 0D114J4, 0D114K4, 0D114Z4, 0D15074, 0D150J4, 0D150K4, 0D150Z4, 0D153J4, 0D15474, 0D154J4, 0D154K4, 0D154Z4, 0D16074, 0D160J4, 0D160J9, 0D160JA, 0D160K4, 0D160K9, 0D160KA, 0D160Z4, 0D163J4, 0D16474, 0D164J4, 0D164J9, 0D164JA, 0D164K4, 0D164K9, 0D164KA, 0D164Z4, 0D16874, 0D168J4, 0D168J9, 0D168JA, 0D168K4, 0D168K9, 0D168KA, 0D168Z4, 0D1B0Z4,
			0D1B4Z4, 0D1B8Z4, 0D1H0Z4, 0D1H4Z4, 0D1H8Z4, 0D1K0Z4, 0D1K4Z4, 0D1K8Z4, 0D1L0Z4, 0D1L4Z4, 0D1L8Z4, 0D1N0Z4, 0D1N4Z4, 0D1N8Z4, 0D20X0Z, 0D20XUZ, 0D20XYZ, 0D787ZZ, 0D7E7ZZ, 0DBB7ZZ, 0DH50DZ, 0DH50UZ, 0DH53DZ, 0DH53UZ, 0DH54DZ, 0DH54UZ, 0DH57DZ, 0DH57UZ, 0DH58DZ, 0DH58UZ, 0DH63UZ, 0DH64UZ, 0DHA3UZ, 0DHA4UZ, 0DHA8UZ, 0DN87ZZ, 0DNE7ZZ, 0DW04UZ, 0DW08UZ, 0WQFXZ2, 3E1G78Z, 3E1H78Z
	Transplantation	996.82,996.86,996.87, V42.7, V42.83, V42.84, 46.97, 50.5, 50.51, 50.59, 52.8, 52.80, 52.82, 52.83, 52.84, 52.85, 52.86	T86.40-T86.42, T86.890, T86.891, T86.899, T86.850, T86.851, T86.859, Z94.4, Z94.82, Z94.83, 0DY80Z0, 0DY80Z1, 0DY80Z2, 0DYE0Z0, 0DYE0Z1, 0DYE0Z2, 0FY00Z0, 0FY00Z1, 0FY00Z2, 0FYG0Z0, 0FYG0Z1, 0FYG0Z2, 3E030U0, 3E030U1, 3E033U0, 3E033U1, 3E0J3U0, 3E0J3U1, 3E0J7U0, 3E0J7U1, 3E0J8U0, 3E0J8U1
**Hematologic or immunologic**	Hereditary anemias	282.0-282.6	D55-D58
	Aplastic anemias	284	D60-D61, D71
	Hereditary immunodeficiency	279.0-279.9, 288.1, 288.2, 446.1	D80-D89, D72.0, M30.3, M35.9
	Coagulation/hemorrhagic	286.0, 286.3, 287.32, 287.33, 287.39	D66, D68.2, D69.41, D69.42, D69.49
	Leukopenia	288.01, 288.02	D70.0, D70.4

© 2025 Odetola F et al. *JAMA Network Open.*

Categories	Subcategories	ICD-9	ICD-10
	Hemophagocytic Syndromes	288.4	D76.1-D76.3
	Sarcoidosis	135	D86.9
	Acquired immunodeficiency	042-044	B20-B24
	Polyarteritis nodosa and related conditions	446.0, 446.21, 446.4-446.7	M30.0, M31.0, M31.1, M31.30, M31.4, M31.6
	Diffuse diseases of connective tissue	710.0, 710.1, 710.3	M32.10, M33.90, M34.0, M34.1, M34.9
	Other	41.5	07TP0ZZ, 07TP4ZZ
	Devices	N/A	N/A
	Transplantation	41.00, 41.01, 41.02, 41.03, 41.04, 41.05, 41.06, 41.07, 41.08, 41.09, 41.94	07YP0Z0, 07YP0Z1, 07YP0Z2, 30230AZ, 30230G0, 30230G1, 30230X0, 30230X1, 30230Y0, 30230Y1, 30233AZ, 30233G0, 30233G1, 30233X0, 30233X1, 30233Y0, 30233Y1, 30240AZ, 30240G0, 30240G1, 30240X0, 30240X1, 30240Y0, 30240Y1, 30243AZ, 30243G0, 30243G1, 30243X0, 30243X1, 30243Y0, 30243Y1, 30250G0, 30250G1, 30250X0, 30250X1, 30250Y0, 30250Y1, 30253G0, 30253G1, 30253X0, 30253X1, 30253Y0, 30253Y1, 30260G0, 30260G1, 30260X0, 30260X1, 30260Y0, 30260Y1, 30263G0, 30263G1, 30263X0, 30263X1, 30263Y0, 30263Y1
**Metabolic**	Amino acid metabolism	270.0-270.9	E70.0, E70.2, E70.3, E70.4, E70.8, E71.0-E71.5, E72.0-E72.4, E72.8, E72.9
	Carbohydrate metabolism	271.0-271.9	E74.0-E74.4, E74.8, E74.9
	Lipid metabolism	272.0-272.9	E75, E77.0, E77.1, E78.0-E78.4, E78.5-E78.9, E88.1, E88.8
	Storage disorder	277.3, 277.5	E76.0-E76.3, E85
	Other metabolic disorders	275.0-275.3, 277.2,277.6, 277.8-277.9	277.4, E79.1, E79.8, E80.4-E80.7, E83.0, E83.1, E83.3, E83.4, D84.1, E88, H49.8
	Endocrine disorders	243, 253.2, 253.5, 253.6, 235.9, 255.0, 255.13, 255.2, 06.4, 06.52, 06.81, 07.3, 07.64, 07.65, 07.68, 07.69, 62.4, 62.41,	E00.9, E23.0, E23.2, E22.2, E23.3, E23.7, E24.0, E24.2, E24.3, E24.8, E24.9, E26.81, E25.0, E25.8, E25.9, 0GT00ZZ, 0GT04ZZ, 0GT40ZZ, 0GT44ZZ, 0GTK0ZZ, 0GTK4ZZ,

© 2025 Odetola F et al. *JAMA Network Open.*

Categories	Subcategories	ICD-9	ICD-10
		64.5, 65.5, 65.51, 65.53, 65.6, 65.61, 65.63, 68.4, 68.41, 68.49, 68.5, 68.51, 68.59, 68.6, 68.61, 68.69, 68.7, 68.71, 68.79	0GTR0ZZ, 0GTR4ZZ, 0UT20ZZ, 0UT24ZZ, 0UT27ZZ, 0UT28ZZ, 0UT2FZZ, 0UT40ZZ, 0UT44ZZ, 0UT47ZZ, 0UT48ZZ, 0UT70ZZ, 0UT74ZZ, 0UT90ZZ, 0UT94ZZ, 0UT97ZZ, 0UT98ZZ, 0UT9FZZ, 0UTC0ZZ, 0UTC7ZZ, 0UTC8ZZ, 0VTC0ZZ, 0VTC4ZZ, 0W4M070, 0W4M0J0, 0W4M0K0, 0W4M0Z0, 0W4N071, 0W4N0J1, 0W4N0K1, 0W4N0Z1
	Devices	V45.85, V53.91, 86.06	Z46.81, Z96.41, 0JH60VZ, 0JH63VZ, 0JH70VZ, 0JH73VZ, 0JH80VZ, 0JH83VZ, 0JHD0VZ, 0JHD3VZ, 0JHF0VZ, 0JHF3VZ, 0JHG0VZ, 0JHG3VZ, 0JHH0VZ, 0JHH3VZ,
			0JHL0VZ, 0JHL3VZ, 0JHM0VZ, 0JHM3VZ, 0JHN0VZ, 0JHN3VZ, 0JHP0VZ, 0JHP3VZ, 0JHT0VZ, 0JHT3VZ
	Transplantation	N/A	N/A
**Other Congenital or Genetic Defect**	Chromosomal anomalies	758.0-758.9	Q90.9, Q91.3, Q91.4, Q91.7, Q92.8, Q93, Q95.0, Q96.9, Q97, Q98, Q99.8, Q99.9
	Bone and join anomalies	259.4, 737.3, 756.0-756.5	E34.3, M41.0, M41.2, M41.30, M41.8, M41.9, M43.30, M96.5, Q72.2, Q75.0, Q75.2, Q75.9, Q76.0-Q76.2, Q76.4-Q76.7, Q77, Q78.0-Q78.4, Q78.8, Q78.9
	Diaphragm and abdominal wall	553.3, 756.6, 756.7	K44.9, Q79.0-Q79.5, Q79.9, Q79.59
	Other congenital anomalies	757.39, 759.7-759.9	Q81, Q87.1-Q87.3, Q87.40, Q87.81, Q87.89, Q89.7, Q89.9, Q99.2
**Malignancy**	Neoplasms	140-209, 230-239, 00.10, 99.25	C00-C96, D01-D09, D3A.0, D37-D49, Q85.0, 3E00X05, 3E01305, 3E02305, 3E03005, 3E03305, 3E04005, 3E04305, 3E05005, 3E05305, 3E06005, 3E06305, 3E0A305, 3E0F305, 3E0F705, 3E0F805, 3E0G305, 3E0G705, 3E0G805, 3E0H305, 3E0H705, 3E0H805, 3E0J305, 3E0J705, 3E0J805, 3E0K305, 3E0K705, 3E0K805, 3E0L305, 3E0L705, 3E0M305, 3E0M705, 3E0N305, 3E0N705, 3E0N805, 3E0P305, 3E0P705, 3E0P805, 3E0Q305, 3E0Q705, 3E0R305, 3E0S305, 3E0V305,

© 2025 Odetola F et al. *JAMA Network Open.*

Categories	Subcategories	ICD-9	ICD-10
			3E0W305, 3E0Y305, 3E0Y705
	Devices	N/A	N/A
	Transplantation	996.85, V42.81, V42.82	T86.00-T86.02, T86.09, Z94.81, Z94.84
**Premature and Neonatal**	Fetal malnutrition	764.01, 764.02, 764.11, 764.12, 764.21, 764.22, 764.91, 764.92	P05.01, P05.11, P05.02, P05.12, P05.2, P05.9
	Extreme immaturity	765.01, 765.02, 765.11, 765.12, 765.21-765.23	P07.01, P07.02, P07.21-P07.25
	Cerebral hemorrhage at birth	767.0	P10.0, P10.1, P10.4, P52.4, P52.8
	Spinal cord injury at birth	767.4	P11.5
	Birth asphyxia	768.5, 768.9	P21.0, P21.9, P84
	Respiratory diseases	770.2, 770.7	P25.0-P25.3, P25.8, P27.0, P27.1, P27.8
	Hypoxic-ischemic encephalopathy	768.7	P91.6
	Other	771.0, 771.1, 772.13, 772.14, 773.3, 773.4, 774.7, 776.5, 777.53, 778.0, 779.7	P35.0, P35.1, P25.21, P25.22, P56.0, P57.0, P57.8, P61.3, P61.4, P77.3, P83.2, P91.2
**Miscellaneous, Not Elsewhere Classified**	Devices	996.4, 996.66, 996.67, 996.9, V46.2 81.00, 81.01, 81.02, 81.03, 81.04, 81.05, 81.06, 81.07, 81.08, 81.09, 81.30, 81.31, 81.32, 81.33, 81.34, 81.35, 81.36, 81.37, 81.38, 81.39, 84.51	T84.019A, T84.029A, T84.039A, T84.049A, T84.059A, T84.069A, T84.099A, T84.498A, T84.119A, T84.129A, T84.199A, T84.498A, T84.50XA, T84.60XA, , T84.7XXA, T86.90-T86.92, T86.99, T86.10-T86.12, T86.40-T86.42, T86.20-T86.22, T86.810, T86.811, T86.819, T86.00-T86.02, T86.09, T86.890, T86.891, T86.899, T86.850, T86.851, T86.859, T86.5, T86.890, T86.891, T86.899, T87.0X9, T87.1X9, T87.2, Y83.1, Y83.3, Z99.81, 0RG00J0, 0RG00J1, 0RG00JJ, 0RG00K0, 0RG00K1, 0RG00KJ, 0RG00Z0, 0RG00Z1, 0RG00ZJ, 0RG03J0, 0RG03J1, 0RG03JJ, 0RG03K0, 0RG03K1, 0RG03KJ, 0RG03Z0, 0RG03Z1, 0RG03ZJ, 0RG04J0, 0RG04J1, 0RG04JJ, 0RG04K0, 0RG04K1, 0RG04KJ, 0RG04Z0, 0RG04Z1, 0RG04ZJ,

© 2025 Odetola F et al. *JAMA Network Open.*

Categories	Subcategories	ICD-9	ICD-10
			0RG10J0, 0RG10J1, 0RG10JJ, 0RG10K0, 0RG10K1, 0RG10KJ, 0RG10Z0, 0RG10Z1, 0RG10ZJ, 0RG13J0, 0RG13J1, 0RG13JJ, 0RG13K0, 0RG13K1, 0RG13KJ, 0RG13Z0, 0RG13Z1, 0RG13ZJ, 0RG14J0, 0RG14J1, 0RG14JJ, 0RG14K0, 0RG14K1, 0RG14KJ, 0RG14Z0, 0RG14Z1, 0RG14ZJ, 0RG40J0, 0RG40J1, 0RG40JJ, 0RG40K0, 0RG40K1, 0RG40KJ, 0RG40Z0, 0RG40Z1, 0RG40ZJ, 0RG43J0, 0RG43J1, 0RG43JJ, 0RG43K0, 0RG43K1, 0RG43KJ, 0RG43Z0, 0RG43Z1, 0RG43ZJ, 0RG44J0, 0RG44J1, 0RG44JJ, 0RG44K0, 0RG44K1, 0RG44KJ, 0RG44Z0, 0RG44Z1, 0RG44ZJ, 0RG60J0, 0RG60J1, 0RG60JJ, 0RG60K0, 0RG60K1, 0RG60KJ, 0RG60Z0, 0RG60Z1, 0RG60ZJ, 0RG63J0, 0RG63J1, 0RG63JJ, 0RG63K0, 0RG63K1, 0RG63KJ, 0RG63Z0, 0RG63Z1, 0RG63ZJ, 0RG64J0, 0RG64J1, 0RG64JJ, 0RG64K0, 0RG64K1, 0RG64KJ, 0RG64Z0, 0RG64Z1, 0RG64ZJ, 0RGA0J0, 0RGA0J1, 0RGA0JJ, 0RGA0K0, 0RGA0K1, 0RGA0KJ, 0RGA0Z0, 0RGA0Z1, 0RGA0ZJ, 0RGA3J0, 0RGA3J1, 0RGA3JJ, 0RGA3K0, 0RGA3K1, 0RGA3KJ, 0RGA3Z0, 0RGA3Z1, 0RGA3ZJ, 0RGA4J0, 0RGA4J1, 0RGA4JJ, 0RGA4K0, 0RGA4K1, 0RGA4KJ, 0RGA4Z0, 0RGA4Z1, 0RGA4ZJ, 0SG00J0, 0SG00J1, 0SG00JJ, 0SG00K0, 0SG00K1, 0SG00KJ, 0SG00Z0, 0SG00Z1, 0SG00ZJ, 0SG03J0, 0SG03J1, 0SG03JJ, 0SG03K0, 0SG03K1, 0SG03KJ, 0SG03Z0, 0SG03Z1, 0SG03ZJ, 0SG04J0, 0SG04J1, 0SG04JJ, 0SG04K0, 0SG04K1, 0SG04KJ, 0SG04Z0, 0SG04Z1, 0SG04ZJ
	Transplantation	996.80, 6.88, 996.89, 00.91, 00.92, 00.93	T86.5, T86.90-T86.92, T86.99, T86.890, T86.891, T86.899

*Feudtner C, Feinstein JA, Zhong W, Hall M, Dai D. Pediatric complex chronic conditions classification system version 2: updated for ICD-10 and complex medical technology dependence and transplantation. *BMC Pediatr.* 2014; 14:199

© 2025 Odetola F et al. *JAMA Network Open.*
