# Supplementary material for: Health Care Resource Use and Costs After Hospitalization With Multiple Organ Dysfunction in Children
Source: JAMA Netw Open. 2025 Jan 29;8(1):e2456246. doi: 10.1001/jamanetworkopen.2024.56246 (PMC11780478; doi:10.1001/jamanetworkopen.2024.56246)
Supplement: Supplement 2. — Data Sharing Statement [file jamanetwopen-e2456246-s002.pdf]

Data Sharing Statement

Odetola. Health Care Resource Use and Costs After Hospitalization With Multiple Organ Dysfunction in Children. *JAMA Netw Open*. Published January 29, 2025. doi:10.1001/jamanetworkopen.2024.56246

Data
Data available: No
